# A new ladybird spider from Hungary (Araneae, Eresidae)

**DOI:** 10.3897/zookeys.494.8676

**Published:** 2015-04-06

**Authors:** Gábor Kovács, István Prazsák, János Eichardt, Gábor Vári, Henrik Gyurkovics

**Affiliations:** 1 Dózsa tér 4., Bordány, H–6795 Hungary; 2 Department of Medical Biology, Faculty of Medicine, University of Szeged, Dugonics tér 13., Szeged H–6720 Hungary; 3 Arachnological Laboratory, University of West Hungary, Károlyi Gáspár tér 4., Szombathely H–9700 Hungary; 4 Information Technology Department, Albert Szent-Györgyi Health Center, University of Szeged, Tisza L. krt. 107., Szeged H–6720 Hungary; 5 Biological Research Centre, Hungarian Academy of Sciences, Temesvári krt. 62., Szeged H–6726 Hungary

**Keywords:** Ladybird spiders, *Eresus*

## Abstract

According to the most recent taxonomic literature, three species of the genus *Eresus* are known in Central Europe, *Eresus kollari*, *Eresus sandaliatus* and *Eresus moravicus*. We recognized a fourth distinctive species from Hungary, which is described as *Eresus hermani* **sp. n.** *Eresus hermani* has an early spring copulation period, females have a light grey (grizzled) cephalothorax due to a heavy cover of lightly colored setae, and an epigyne with large flat areas posterior to the epigynal pit, while males are distinguished by a broad and blunt terminal tooth of the conductor. An updated and modified comparative table of Řezáč et al. (2008) to include all four Central European *Eresus* species, and a simple key to the species group’s species are given. Habitus, epigyne, vulva and conductor of *Eresus kollari*, *Eresus moravicus* and *Eresus sandaliatus* are also illustrated. An annotated list of papers illustrating *Eresus hermani* due to misidentifications is presented.

## Introduction

The velvet spiders (family Eresidae) are among the most attractive spiders in Europe. The family contains nine genera and 96 described species worldwide. The genus *Eresus* Walckenaer, 1805 contains 15 valid species from Europe, Africa and Asia, of which nine occur in Europe (World Spider Catalog 2015).

According to the latest studies (Řezač et al. 2008, Miller et al. 2012) three species of the *Eresus sandaliatus* group, *Eresus kollari* Rossi, 1846, *Eresus sandaliatus* Martini & Goeze, 1778 and *Eresus moravicus* Řezáč, 2008, occur in Central Europe.

The long and complicated scientific history of the *Eresus sandaliatus* group *sensu* Miller et al. (2012) is discussed in detail in Řezač et al. (2008), so only the Hungarian perspective is described here. The nomenclatural chaos is well illustrated by the fact that *Eresus cinnaberinus* might become valid, possibly as a senior synonym of *Eresus kollari* (Azarkina and Trilikauskas 2012).

The Hungarian spider fauna was first studied in detail by Ottó Herman, who also gave a detailed description of the *Eresus* genus (Herman 1879). Herman indicated the presence of two species, *Eresus ruficapillus* C. L. Koch, 1846 (regarded as misidentification of *Eresus moravicus* by Řezač et al. 2008 due the “reddish-yellowish hairs on the female”) and *Eresus kollari* (as *Eresus cinabarinus* Olivier), distinguishing *α*, *β*, and *γ* color variants, the latter corresponding to *Eresus moravicus*.

However, subsequent authors (e. g. Chyzer and Kulczynski 1918, Samu and Szinetár 1999) recognized only one species, *Eresus cinnaberinus*, with adults during the autumn.

Loksa (1969) mentioned a color form of female *Eresus* (Eresus niger var. ruficapillatus C.L. Koch) from the Mecsek hills and from the vicinity of lake Balaton, which has yellowish hairs on the carapace front, later identified as *Eresus moravicus* by Řezač et al. (2008).

Recently, Řezáč et al. (2008) considered *Eresus cinnaberinus* as *nomen dubium* [but see personal communication of Řezáč referred to in Azarkina and Trilikauskas (2012) as it might not] and proposed the name *Eresus kollari* Rossi, 1846 as valid. In this revision a distinct new species, *Eresus moravicus* was described (Řezač et al. 2008).

*Eresus cinabarinus* γ-color variant of Herman (1879), *Eresus ruficapillus* C.L. Koch and Eresus niger var. ruficapillatus (in Loksa 1969) were all identified as *Eresus moravicus* by Řezač et al. (2008). This means two Hungarian *Eresus* species, *Eresus moravicus* with a late spring–early summer copulation period, and *Eresus kollari* with populations mating in autumn (Kovács et al. 2010).

During an ongoing project aimed at mapping the distribution of Eresidae in Hungary, the presence of an *Eresus* species was observed with an early spring copulation period, which has unique morphological characters, and is described here as new to science.

## Materials and methods

Specimens were either collected individually or by using pitfall traps, and stored in 70% ethyl-alcohol.

We studied 31 males, 15 females and 6 juveniles of *Eresus kollari*; 20 males, 25 females and 4 juveniles of *Eresus hermani* sp. n., and 19 males, 11 females and 3 juveniles of *Eresus moravicus*, and 2 males, 3 females and 2 juveniles of *Eresus sandaliatus*. All the measurements are given in millimeters (mm).

All specimens of the new species examined, including holotype and four paratypes, have been deposited in the Soil Zoological Collection (former Arachnoidea Collection) of the Department of Zoology, Hungarian Natural History Museum (HNHM) Budapest (curator Dr. László Dányi).

Specimens and copulatory organs were studied using a Leica MZ FL III stereomicroscope and photographed by Canon Q Imaging Micro 5.0 RTV at the Institute of Genetics, BRC. Scanning electron micrographs were taken with a Hitachi S-4700 microscope at the Department of Applied and Environmental Chemistry, University of Szeged, Hungary.

### Abbreviations

Standard abbreviations of morphological terms follow Miller et al. (2012). Further abbreviations: PME = posterior median eyes, PLE = posterior lateral eyes, Fe = femur, Pt = patella, Ti = tibia, Ta = tarsus, Mt = metatarsus, ML = median lobe of epigyne, L = leg, juv. = juvenile.

HNHM Hungarian Natural History Museum, Budapest;

NHMW Naturhistorisches Museum, Wien;

PPI Plant Protection Institute of the Hungarian Academy of Sciences, Budapest;

JLPC private collection of Jørgen Lissner;

WPPC private collection of Walter Pfliegler.

Translation of Hungarian geographical names in the description of collection material is -*hegy*: hill; -*völgy*: valley.

## Results

### Taxonomy

#### Eresus hermani sp. n.

Taxon classificationAnimaliaAraneaeEresidae

http://zoobank.org/CE9C2B06-FBAC-4246-BD75-EC716F94C34F

**Figure 1.**
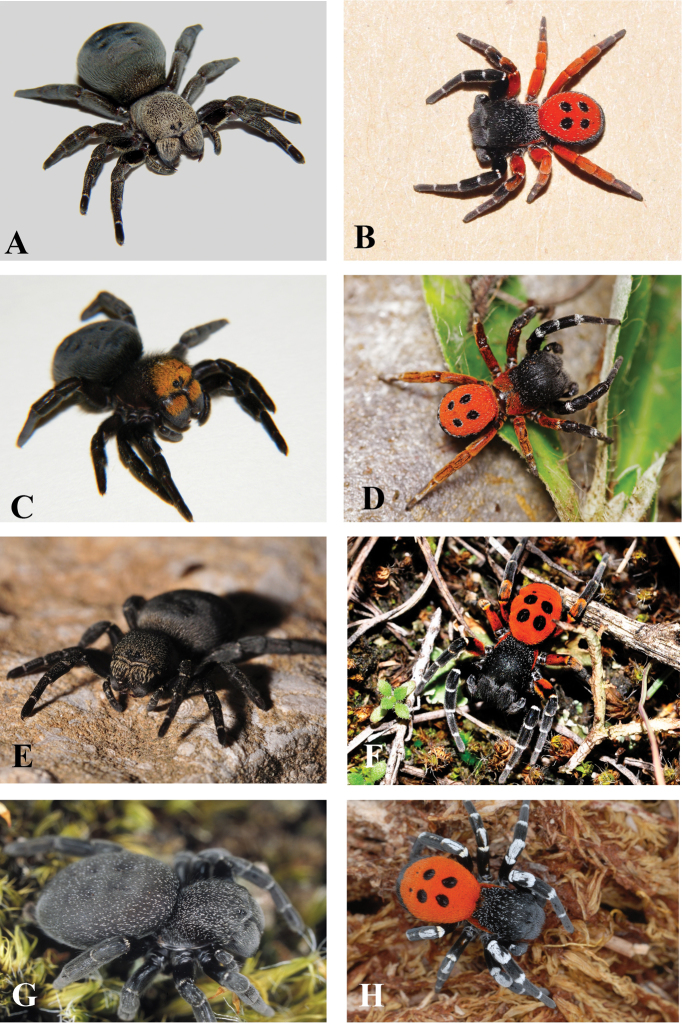
**A–H** Habitus of living *Eresus* species, photographs: **A–B** *Eresus hermani* **A** female (Remete-hegy, Budapest, Hungary) **B** male (Farkas-hegy, Budaörs, Hungary) **C–D** *Eresus moravicus* **C** female (Misina-hegy, Pécs, Hungary) **D** male (Dürnstein, Austria) **E–F** *Eresus kollari* **E** female (Paloznak, Hungary) **F** male (Kéleshalom, Hungary) **G–H** *Eresus sandaliatus* **G** subadult female (near to Silkeborg Langsø, Enebærbakken, Denmark) **H** male (Nørlund, Hallundbæk Stream, Denmark) (**D** courtesy of Walter Pfliegler **G–H** courtesy of Jørgen Lissner).

Figs 1A–B , 3A–C , 4A–B , 5A–B , 6A–B , 7A

Eresus cinnaberinus Szinetár 2006 p 22 fig. 3 (misidentified)
Eresus kollari Kovács et al. 2010 fig. 1C–F, 2D (misidentified)
Eresus kollari Miller et al. 2012 fig. 2A (misidentified)
Eresus kollari Szinetár et al. (2012): table 2, figure 6 (misidentified)

##### Material examined.

**Holotype:** Female – HUNGARY, Budapest, Remete-hegy, N 47°32'26.3", E 19°00'24.1", singled, 23.04.2011., G. Kovács (HNHM, collection number: HNHM Araneae 7612).

**Paratypes:** 2 females – HUNGARY, Budapest, Sas-hegy, N 47°28'47.2", E 19°01'04.4", singled, 02.10.2013., G. Kovács, H. Gyurkovics, G., Vári, A. Rákóczi (HNHM, collection number: HNHM Araneae-7630-31). – 2 males HUNGARY, Budapest, Remete-hegy, N 47°32'26.3", E 19°00'24.1", singled, 23.04.2011., G. Kovács, (HNHM, collection number: HNHM Araneae: 7632–33).

##### Remark.

The genus *Eresus* in Central Europe has a long and difficult nomenclatural history. Some available “old names” were examined, such as *Eresus illustris* (presently considered *nomen dubium*, specimens are irretraceable), which is marked as possibly Hungarian (despite the fact Koch himself wrote “Vaterland: Unbekannt” [trans. Locality: Unknown]), but discarded it on the basis of the description and color image (Koch 1838, fig. 317), where the male has six black dots on the opisthosoma and only the dorsal side of hind femora as red, whereas *Eresus hermani* males have only four dots and clearly red hind legs patellae and tibiae, without any black, and tarsi and metatarsi are brownish grey (Fig. 1B). The female of *Eresus illustris* is unknown. The other possible candidate, *Eresus fulvus* Rossi 1846 (type specimens can no longer be found in NHMW), described by female specimens only, can also be excluded as a potential synonym, since they all have a large area covered by yellow/orange setae on the cephalothorax [“nitide fulvus” in the description of Rossi (1846)], whereas *Eresus hermani* females have no truly yellow setae on the prosoma at all; instead, its dorsal cephalothorax is light brownish-grey overall. According to Řezáč et al. 2008 (page 275.) *Eresus fulvus* Rossi differs from *Eresus moravicus* by “having spermatheca that are less lobed, and having copulatory ducts that are almost horizontal in the centre of the vulva.” By contrast, spermathecae of *Eresus hermani* are rather conspicuously lobed, at least as much as in *Eresus moravicus* (Figs 4C, F and 5B, D).

##### Etymology.

Dedicated to Ottó Herman (1835–1914), the Hungarian arachnologist and polymath, who first recognized color variants within Hungarian *Eresus* forms, to commemorate the 100^th^ anniversary of his passing.

##### Diagnosis.

Females of this species differ from all other *Eresus* females by the carapace’s short, off-white to light brown hairs, intermingled with small clumps of long, black hairs, giving a light, grizzled appearance to the prosoma, and by an epigyne with a pair of flat plateaus adjoining the sides of the broad median lobe laterally. Males are characterized by the narrow groove and blunt, broad terminal tooth of the conductor, and distinguished from other *Eresus* species, except *Eresus moravicus*, by having almost entirely red hind legs. They differ from *Eresus moravicus* males by having red color on the thoracic dorsum only laterally, having a less prominent cephalic region with an almost flat area between PLE and PME, and by narrower strips of white setae on L I. This species has an early spring copulation period, and exhibits a marked difference in the sizes of the sexes: males are relatively small, while females are comparatively large among Central European *Eresus* spp. (Table 1).

**Table 1.** Distinguishing morphological characters of species belonging to *Eresus sandaliatus* group (in part after Řezáč et al. 2008).

	*Eresus kollari* Rossi, 1846 morphotype	*Eresus sandaliatus* Martini & Goeze, 1778
**Females**		
Prosoma length	3.6–6.1 (mean 4.7)	4.2–7.2 (mean 5.4)
Color of prosoma	black, sparsely sprinkled with off-white to light brown setae, more heavily anteriorly (Fig. 1E)	black, sparsely sprinkled with off-white to light brown setae, more heavily anteriorly (Fig. 1G)
Epigyne	(i) epigynal pit extending all the way to posterior epigyne (Figs 4G, H, 5E)	(i) epigynal pit extending all the way to posterior epigyne (Figs 4J, K, 5G)
	(ii) anterior 1/3 of fissures markedly incurvated sidewards, anterior tip usually not incurvated (Figs 4G,H, 5E)	(ii) anterior 1/3 of fissures slightly inclined sideways, anterior tip weakly bent (Figs 4J, K, 5G)
Vulva	(i) anterior section of copulatory ducts strongly sclerotized, usually elongated (Figs 4I, 5F)	(i) anterior section of copulatory ducts weakly sclerotized, usually globular Figs 4L, 5H)
	(ii) spermathecae distinctly lobed (Figs 4I, 5F)	(ii) spermathecae indistinctly lobed (Figs 4L, 5H)
Approximate ratio between greatest width of ML and that of epigyne	4:10	5:10
**Males**		
Prosoma length	2.6–4.2 (mean 3.6)	2.9–4.1 (mean 3.6)
Number of black spots on opisthosoma	usually 4	usually 6
White hairs on opisthosoma	usually present	usually absent
Color of hind legs	proximally red, distally black (Fig. 1F)	black, exceptionally with some red on femur (Fig. 1H)
White transverse stripes on Leg I–II	narrow, covering only the distal edge of segments (Fig. 1 F)	very broad at the distal part of segments, widely extending into the proximal part of next segment (Fig. 1H)
Red color on thoracic dorsum	only on flanks, at most a few red hairs posteriorly (Fig. 1F)	only on flanks, at most a few red hairs posteriorly (Fig. 1H)
Conductor in lateral view	moderately wrinkled, much longer than wide (Fig. 3 H)	almost smooth, about as long as wide (Fig. 3K)
Terminal tooth of conductor	small, almost straight, pointed (Figs 3G, H, I)	strong, long, almost straight, tip cropped (Fig. 3J, K, L)
Groove of conductor in lateral view	shallow, V-shaped (Fig. 3 H.)	deep, U-shaped (Fig. 3K.)

	*Eresus hermani* sp. n.	*Eresus moravicus* Řezáč, 2008
**Females**		
Prosoma length	6.6–9.9 (mean 8.2)	5.9–9.9 (mean 7.5)
Color of prosoma	entire prosoma grizzled light brown due to a heavy cover of off-white to light brown setae (Fig. 1A)	black, except orange anterior (Fig. 1C)
Epigyne	(i) flat plateaus between the posterior edge of epigynal pit and posterior of epigyne at sides of median lobe (Figs 4A, B, 5A, 6A)	(i) epigynal pit extending all the way to posterior epigyne (Figs 4D, 4E, 5C)
	(ii) anterior ½ of fissures parallel to midline, anterior tip strongly incurved (Figs 4A, B, 5A, 6A)	(ii) anterior ½ of fissures slightly diverging laterally, anterior tip strongly incurved–*see note* (Figs 4D, 5E, 5C)
Vulva	(i) anterior section of copulatory ducts weakly sclerotized, usually globular (Figs 4B, 5B, 6B)	(i) anterior section of copulatory ducts strongly sclerotised, usually elongated (Figs 4D, 5D)
	(ii) spermathecae strongly lobed (Figs 4C, 5B)	(ii) spermathecae strongly lobed (Figs 4D, 5B)
Approximate ratio between greatest width of ML and that of epigyne	6:10	5:10
**Males**		
Prosoma length	2.9–4.1 (mean 3.4)	3.5–5.6 (mean 4.6)
Number of black spots on opisthosoma	nearly always 4	nearly always 4
White hairs on opisthosoma	nearly always present	nearly always present
Color of hind legs	red, tarsal joints brownish grey (Fig. 1B)	red, tarsal joints brownish grey (Fig. 1D)
White transverse stripes on Leg I–II	narrow, covering only the distal edge of segments (Fig. 1B)	broad at the distal part of segments, usually extending to the proximal end of next segment (Fig. 1D)
Red color on thoracic dorsum	only on flanks, at most a few red hairs posteriorly (Fig. 1B)	extends to the middle, at least posteriorly (Fig. 1D)
Conductor in lateral view	wrinkled, clearly wider than long (Fig. 3B)	wrinkled, somewhat longer than wide (Fig. 3E)
Terminal tooth of conductor	strongly incurvated, broad and blunt (Fig. 3B, C)	strongly incurvated, narrows to a relatively pointed tip (Fig. 3E, F)
Groove of conductor in lateral view	deep, narrow, **ν** (Greek nu) or narrow U shaped (Fig. 3B.)	round (Fig. 3E.)

**Note:** Without exception, the epigyne of *Eresus moravicus* specimens that we studied match those in Fig. 2L–P in Řezáč et al. (2008), but differ slightly from that shown in Fig. 2K (Řezáč et al. 2008), which seems to be depicted also as a drawing in Fig. 4H (Řezáč et al. 2008). The main difference is the direction of the anterior portion of fissures, which are typically directed slightly laterally, instead of medially. To aid differentiation of *Eresus moravicus*, we provide comparative photographs and a drawing of *Eresus moravicus* epigyne in Fig. 4D–E and Fig. 5C, which we believe to be typical of the species.

##### Description.

**Male. Prosoma** (Fig. 1B): Length 2.9–4.1 (mean 3.4, N = 15) Prominent, color dark ferruginous brown, covered by long, black hairs intermingled with scattered, short, white ones. Cephalic region barely broader than thoracic part, weakly broadening towards the front, steeply raised posteriorly, but area between PME and PLE nearly flat. Thoracic part bordered laterally by narrow red stripes, never extending to posterior dorsum.

**Chelicerae:** Blackish-brown, covered by long, nearly adpressed black hairs; basal half with scattered white hairs on the front.

**Legs:** Legs I–II dark orange-brown with black hairs; Fe II and Pt II orange with red hairs, Ti II often with a dorsal patch of red hairs. Distal edges of Fe, Pt, Ti and Ta with narrow, white stripe dorsally, usually not extending to the proximal part of the next distal segment. Legs III and IV largely orange, covered with red hairs, Ta and Mt dull grayish-brown due to a mixture of reddish and black hairs, except for a proximo-dorsal patch of red on Mt.

**Opisthosoma** (Fig. 1B): Dorsally red with scattered white hairs except for two pairs of black spots. Red area and black spots seamed by a more-or less continuous line of white hairs. Ventral side of opisthosoma black with the exception of some red hairs on the branchial opercula.

**Palps** (Fig. 3A–C): Conductor broad, strongly wrinkled. Terminal tooth broad and blunt, somewhat longer than the lamella, with a strong, sudden bend at the base or somewhat more distally. Groove deep, narrow, **ν** (Greek nu) or narrow U shaped at the base in lateral view. Inner, spiny lamella high, about as high as terminal tooth.

**Female. Prosoma** (Fig. 1A): Length 6.6–9.9 (mean 8.2, n = 21), prominent, especially the cephalic region, dark orange-brown with a heavy cover of short, off-white to light brown hairs and with scattered, small clumps of long, black hairs giving a grizzled appearance.

**Chelicerae:** Dark orange brown, front of basal 1/3–3/4 same color as prosoma.

**Legs:** Rusty red, Fe, Pt, Ti and Mt of all legs covered by black hairs with pale brown hairs scattered among them, the latter gradually decreasing in number from L I to L IV, usually clustering to form indistinct cross bands dorsally at the distal edge of each segments. Ta usually black, except for a small cluster of pale hairs basally.

**Palps:** Similar in color to L I.

**Opisthosoma** (Fig. 1A): Brownish-black, covered by long black hairs with a scattering of short pale hairs at its anterior.

**Epigyne** (Figs 4A, 5A, 6A): Moderately deep, median lobe broad (ratio between the greatest width of ML to the greatest width of epigyne: 6:10), considerably flared posteriorly, reaching well over the posterior margin of the epigynal pit. Posterior edge of the epigynal depression not reaching posterior epigyne, but followed by a pair of flat, somewhat wrinkled plateaus adjoining the fissures laterally. Posterior part of fissures inclined towards the midline, turning parallel to the longitudinal axis before the short, incurved anterior tips.

**Vulva** (Figs 4B, 5B, 6B): Spermathecae distinctly lobed, reaching further laterally than copulatory ducts. Anterior part of copulatory ducts weakly sclerotized, usually circular, exceptionally elongated in outline.

##### Simplified key to the species of the *Eresus sandaliatus* group

**Females**

**Table:** 

1	Anterior of cephalic region covered by bright yellow/orange setae	***Eresus moravicus***
–	No bright yellow/orange setae on prosoma	**2**
2	Entire prosoma covered heavily by off-white to light brown setae; large	***Eresus hermani* sp. n.**
–	Prosoma sparsely sprinkled with lightly colored setae, somewhat more heavily on the front; smaller	**3**
3	Anterior of fissures only slightly inclined sideways, as in Fig. 5G, spermathecae indistinctly lobed, anterior of copulatory ducts nearly round in outline, weakly sclerotized	***Eresus sandaliatus***
–	Anterior of fissures markedly incurvated sideways, as in Fig. 5F, spermathecae distinctly lobed, anterior of copulatory ducts elliptical, strongly sclerotized.	***Eresus kollari***

**Males**

**Table:** 

1	Terminal tooth of conductor strongly incurvated, hind legs almost entirely red	**2**
–	Terminal tooth of conductor nearly straight, at most weakly bent, red areas on hind legs not so extensive or entirely absent	**3**
2	Conductor with a blunt terminal tooth and a narrow groove, prosoma barely broadens towards front	***Eresus hermani* sp. n.**
–	Conductor with a pointed terminal tooth and a round groove, prosoma strongly broadens towards front	***Eresus moravicus***
3	Conductor with a strong, long and slightly bent terminal tooth and a U-shaped (in lateral view) groove, hind legs nearly devoid of red setae	***Eresus sandaliatus***
–	Conductor with a short, straight terminal tooth and a V-shaped (in lateral view) groove, hind legs with extensive areas of red color	***Eresus kollari***

##### Distribution.

**Figure 2.**
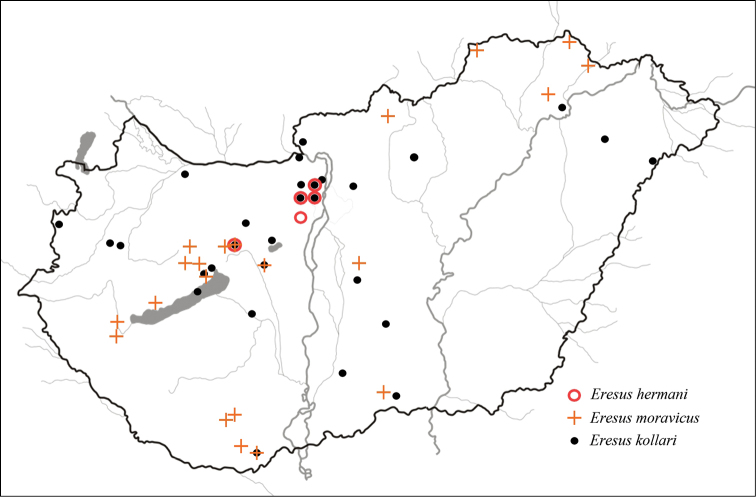
Known localities of all three *Eresus* species occurring in Hungary.

Known from seven localities (Fig. 2): Budapest: Remete-hegy (*locus typicus*), Mátyás-hegy, Sas-hegy, Budaörs: Farkas-hegy, Érd: Fundoklia-völgy and Várpalota-Inota: Víztározó, Baglyas-hegy. With the exception of Érd: Fundoklia-völgy, *Eresus hermani* proved to be syntopic with *Eresus kollari*, whereas all three *Eresus* sp. occurring in Hungary, *Eresus hermani*, *Eresus kollari* and *Eresus moravicus* are syntopic at Várpalota-Inota: Baglyas-hegy.

**Figure 3.**
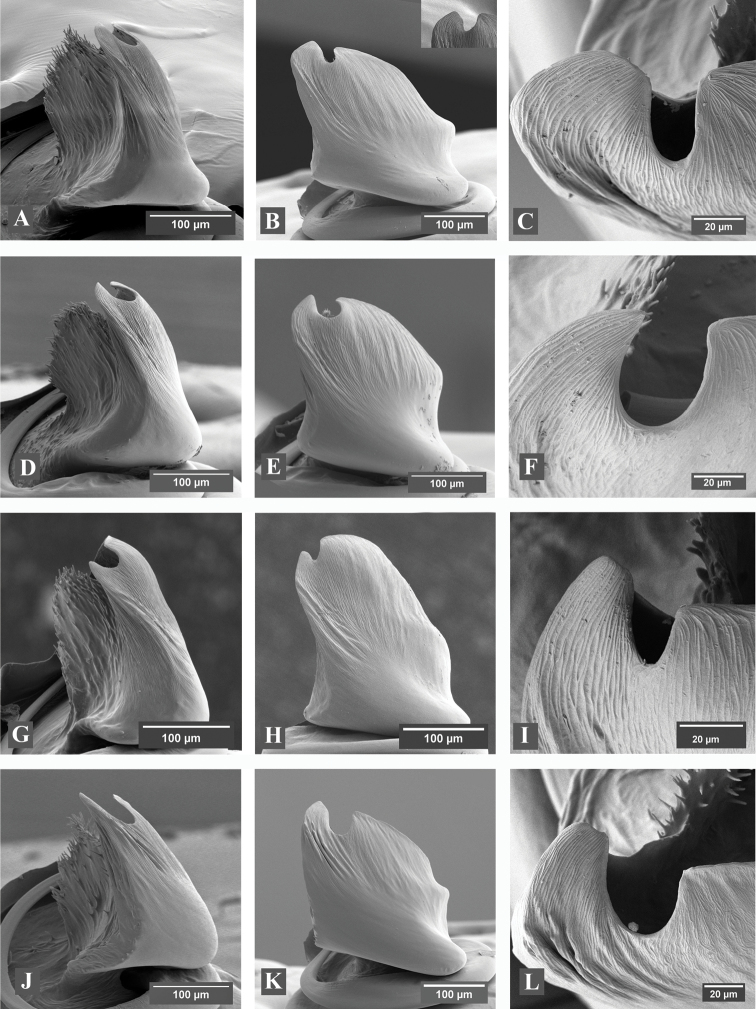
**A–L** Scanning electron micrographs of *Eresus* male palps: **A–C** *Eresus hermani* (Sas-hegy, Budapest, Hungary) **D–F** *Eresus moravicus* (Örkény-Táborfalva-Tatárszentgyörgy, Hungary) **G–I** *Eresus kollari* (Farkas-hegy, Budaörs, Hungary) **J–L** *Eresus sandaliatus* (Aulum, Denmark) **A, D, G, J** ventral **B, E, H, K** lateral and **C, F, I, L** apical view; inset in **B:** a variant of conductor tip with unusually wide groove (Sas-hegy, Budapest, Hungary).

**Figure 4.**
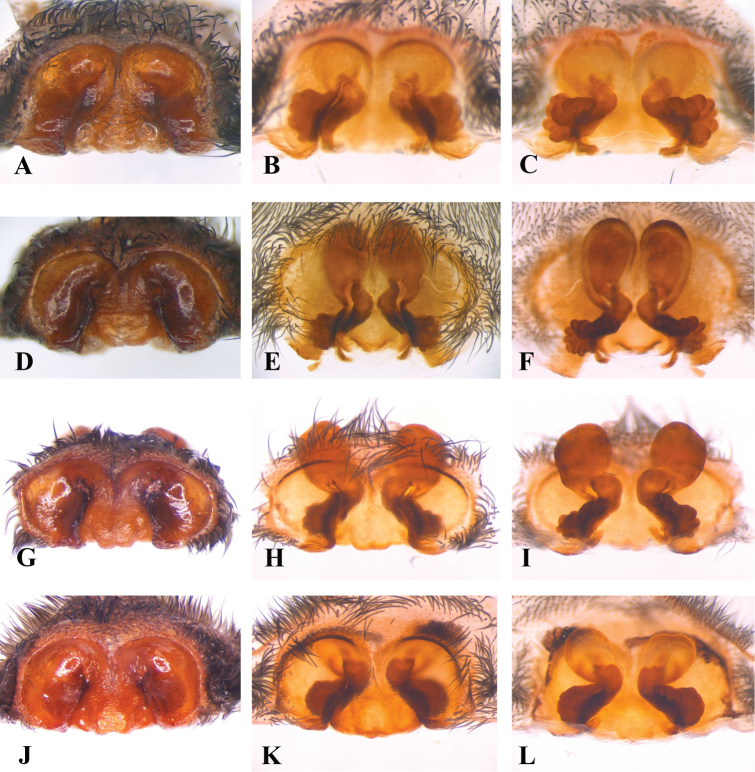
**A–L** Copulatory organs of *Eresus* adult females: **A–C** *Eresus hermani* (Sas-hegy, Budapest, Hungary) **D**–**F** *Eresus moravicus* (**D** Misina-hegy, Pécs, Hungary **E–F** Dürnstein, Austria) **G–I** *Eresus kollari* (Farkas-hegy, Budaörs, Hungary) **J**–**L** *Eresus sandaliatus* (near to Tranemose moor Northwest Jutland, Denmark) **A, D, G, J** epigyna **B, E, H, K** epigyna* **C, F, I, L** vulvae* (*: macerated).

**Figure 5.**
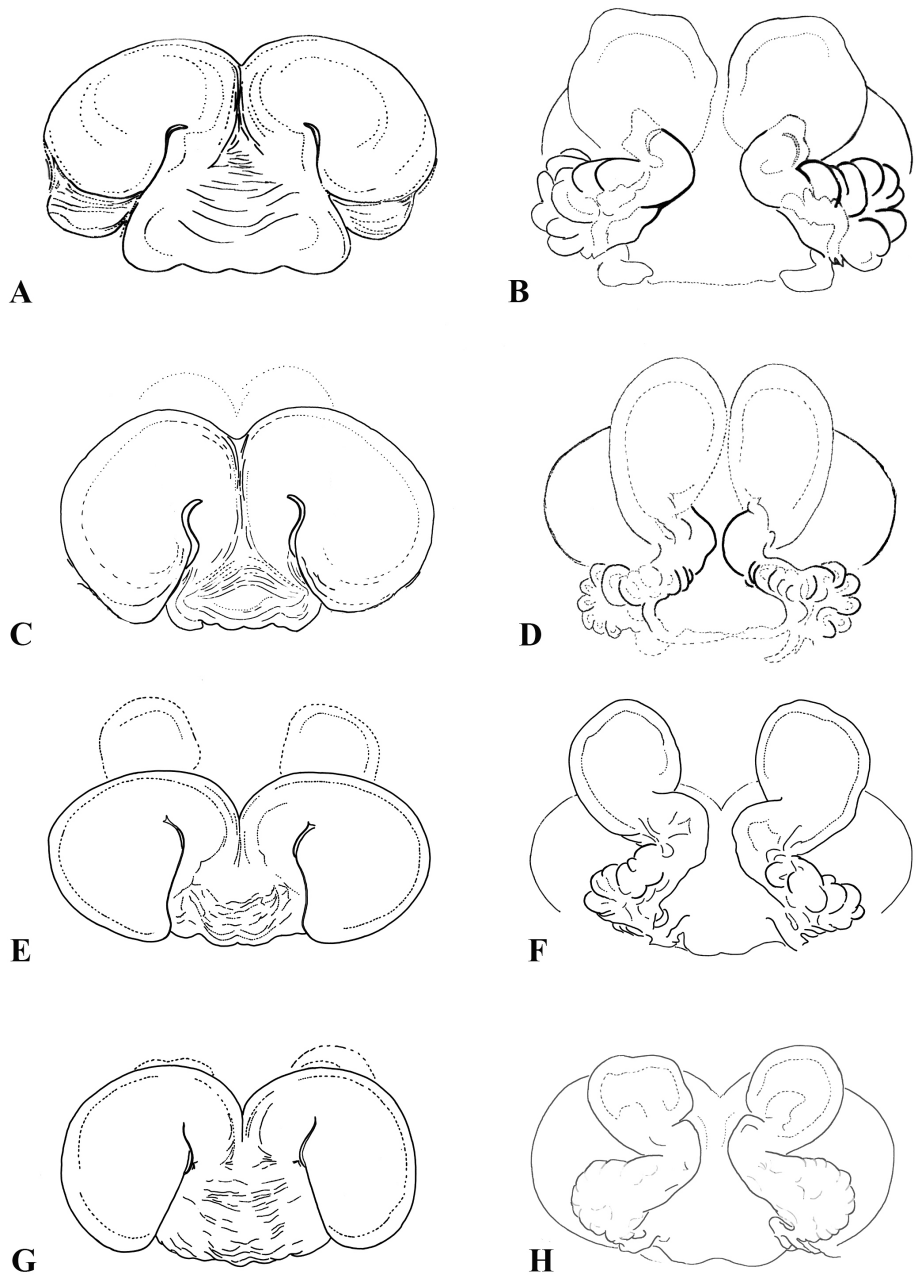
**A–H** Schematic drawings of *Eresus* female copulatory organs: **A–B** *Eresus hermani* (Sas-hegy, Budapest, Hungary) **C–D** *Eresus moravicus* (Dürnstein, Austria) **E–F** *Eresus kollari* (Farkas-hegy, Hungary) **G–H** *Eresus sandaliatus* (near Tranemose moor, Northwest Jutland, Denmark) **A, C, E, G** epigyna **B, D, F, H** vulvae.

**Figure 6.**
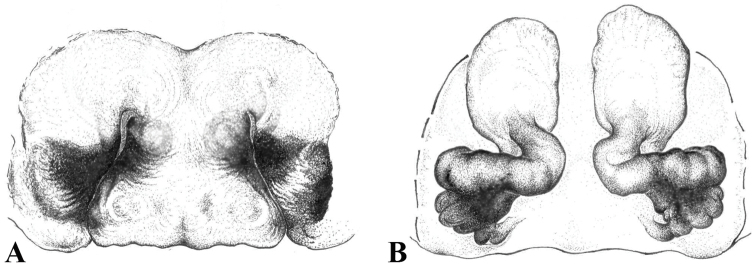
Drawings of *Eresus hermani* female copulatory organ, rare variant (Fundoklia-völgy, Érd, Hungary): **A** epigyne **B** vulva. Note the rounded anterior edge of the plateaus lateral to the median lobe in **A** and the elongated copulatory duct in **B.**

**Figure 7.**
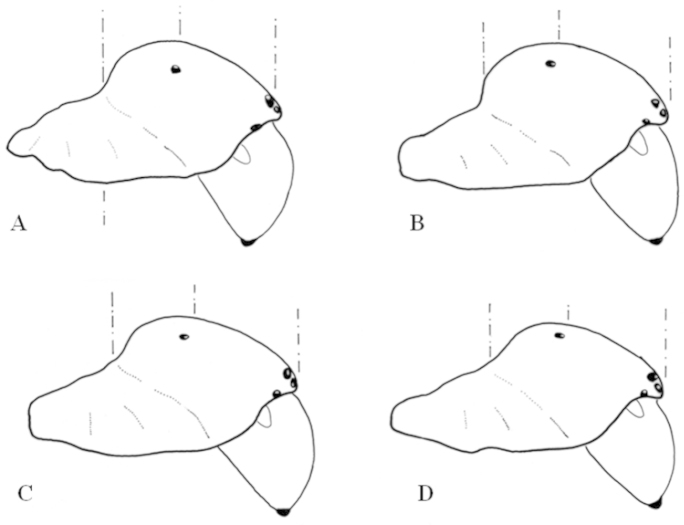
Outline of male prosomas of *Eresus* spp. belonging to the *Eresus sandaliatus* group, in lateral view **A** *Eresus hermani* **B** *Eresus moravicus* **C** *Eresus kollari* **D** *Eresus sandaliatus* (**B, C, D** after Fig. 4. of Řezáč et al. 2008).

##### Habitat.

Edges of a local variety of downy oak scrub woodland (*Ceraso mahaleb-Quercetum pubescentis*) and the interim zone between calcareous open rocky grasslands (*Seselio leucospermi-Festucetum pallentis*) and degraded scrubland.

##### Phenology.

*Eresus hermani* matures in August-September, wandering males can be found from the end of March to the end of April (inferred copulation period) and females lay eggs in June. This phenology clearly sets *Eresus hermani* apart from the other Hungarian *Eresus* species: *Eresus moravicus* matures in late spring and mates in early summer, while *Eresus kollari* matures in late summer – early autumn, immediately followed by a copulation period in autumn. The phenology of *Eresus hermani* is essentially the same as that of *Eresus sandaliatus* (Řezač et al. 2008), which, however, does not occur in Hungary or within the Carpathian Basin.

##### Additional material examined.

**Hungary:** Remete-hegy, Budapest (1 ♀, 01.11.2008., G. Kovács, HNHM Araneae-7669); Remete-hegy, Budapest (1 ♀, 02.09.2008., G. Kovács, HNHM Araneae-7670); Remete-hegy, Budapest (3 ♀, 2 ♂, 05.04.2008., G. Kovács, HNHM Araneae-7671); Remete-hegy, Budapest (1 ♀, 1 juv., 18.04.2008., G. Kovács, HNHM Araneae-7672); Farkas-hegy, Budaörs (1 ♀, 22.09.2013., G. Kovács, H. Gyurkovics, G. Vári, D. V. Nagy, HNHM Araneae-7673); Farkas-hegy, Budaörs (2 ♂, 14.04.2013., H. Gyurkovics, G. Vári, HNHM Araneae-7674; Sas-hegy, Budapest (4 ♂, 07.04.2012., A. Rákóczi, HNHM Araneae-7675); Sas-hegy, Budapest (4 ♂, 25.03.2012., A. Rákóczi, HNHM Araneae-7676); Remete-hegy, Budapest (1 ♂, 16.04.2005., G. Kovács, HNHM Araneae-7677; Farkas-hegy, Budaörs (1 ♂, 13.04.2012., G. Kovács, HNHM Araneae-7678); Farkas-hegy, Budaörs (1 ♂, 21.04.2010., J. Bodor, HNHM Araneae-7679); Remete-hegy, Budapest (5 ♀, 16.09.2012., G. Kovács, HNHM Araneae-7680); Remete-hegy, Budapest (1 ♀, 28.09.2008., G. Kovács, HNHM Araneae-7681); Remete-hegy, Budapest (3 ♀, 23.04.2011., G. Kovács, HNHM Araneae-7682); Remete-hegy, Budapest (1 ♀, 31.03.2011., G. Kovács, HNHM Araneae-7683); Sas-hegy, Budapest (6 ♀, 02.10.2013. H. Gyurkovics, A. Rákóczi, G. Vári, HNHM Araneae-7684); Érd, Fundoklia-völgy (1 ♀, 02.10.2013. G. Vári, HNHM Araneae-7685-86); Érd, Fundoklia-völgy, (1 ♀, 02.10.2013., G. Kovács, HNHM Araneae-7687); Várpalota-Inota (2 juv., 06.07.2014., G. Kovács, G. Vári, HNHM Araneae-7688), Mátyás-hegy, Budapest (5 ♂, 1933, G. Kolosváry, HNHM Araneae-2943).

##### Remarks on misidentifications.

Cs. Szinetár (2006): p. 23. Fig. 3

The caption of this figure says "Female *Eresus cinnaberinus*", but, in fact, the picture shows a female *Eresus hermani* sp. n., as is evident from the heavy cover of light setae on the prosoma and the base of chelicerae.

Kovács et al. (2010): figure 1C–F figure 2D

According to captions, fig. 1C–F of this paper depict the genital organs of female *Eresus kollari*. However, the anterior part of fissures of the epigyna are nearly parallel, epigynal pits are followed by large flat plateaus at the sides of median lobes, anterior copulatory ducts are round and weakly sclerotized, spermathecae strongly lobed, all features that distinguish *Eresus hermani* sp. n. unambiguously. Additionally, the epigyne shown in fig. 1E is grossly malformed, having supernumerary rudiments of fissures, a kind of abnormality frequent among females raised in captivity. Figure 2D is labeled as female *Eresus kollari*. Again, this figure shows a female *Eresus hermani* sp. n., as evidenced by the dense cover of lightly colored setae on the cephalic region and basal segments of chelicerae. The reason for these misidentifications is that at the time of writing, the authors (including the corresponding author of the present paper) considered females of *Eresus hermani* sp. n. as merely an extreme local variant of *Eresus kollari*. (Note: by contrast, fig. 2F. indeed shows a female *Eresus kollari* next to a male of the same species, as can be judged by the sparsely distributed light setae on the prosoma.)

Miller et al. (2012): figure 2A

Figure 2. A. of this paper is mislabeled as *Eresus kollari*, whereas in fact it depicts a female *Eresus hermani* sp. n. Again, the true identity of the specimen shown in this picture is revealed by the light color of the prosoma and basal chelicerae. The obvious reason for the misidentification is that at the time of the completion of this Atlas, the concept of *Eresus hermani* sp. n. as a discreet species was not yet formed.

Szinetár et al. (2012): table 2, figure 6

In this paper, figure 6. shows a female *Eresus hermani* sp. n. mislabeled as *Eresus kollari*. Heavy cover of the prosoma by lightly colored hairs gives away the identity of the depicted specimen.

## Supplementary Material

XML Treatment for Eresus hermani

## Appendix

**Comparative materials examined**

*Eresus kollari*. **Hungary:** Remete-hegy, Budapest (1 ♀, 19.09.2004., G. Kovács, HNHM Araneae-7689); Remete-hegy, Budapest (4 juv., 25.09.2005., G. Kovács, HNHM Araneae-7690); Remete-hegy, Budapest (2 ♂, 1 juv., 17.09.2006., G. Kovács, HNHM Araneae-7691); Remete-hegy, Budapest (1 ♀, 1 juv., 16.09.2007., G. Kovács, HNHM Araneae-7692); Remete-hegy, Budapest (3 ♀, 2 ♂, 03.10.2007., G. Kovács, HNHM Araneae-7693); Remete-hegy, Budapest (4 ♂, 02.09.2008., G. Kovács, HNHM Araneae-7694); Remete-hegy, Budapest (2 ♀, 3 ♂, 28.09.2008., G. Kovács, HNHM Araneae-7695); Kéleshalom (1 ♂, 14.11.2010., H. Gyurkovics, G. Vári, HNHM Araneae-7696); Remete-hegy, Budapest (1 ♀, 02.04.2011., G. Kovács, HNHM Araneae-7697); Budakalász (3 ♂, 01.09.2011., I. Hahn, L. Somay, HNHM Araneae-7698); Remete-hegy, Budapest (1 ♀, 09.04.2011., G. Kovács, HNHM Araneae-7699); Győrszentiván-Gönyü, Héricses (1 ♀, 28.11.2012., Cs. Szinetár, HNHM Araneae-7700); Farkas-hegy, Budaörs (2 ♀, 4 ♂, 15.09.2013., G. Kovács, HNHM Araneae-7701); Győrszentiván (1 ♂, 30.09.2013., P. Kovács, HNHM Araneae-7702); Sas-hegy, Budapest (3 ♂, 22.09.2010., E. Botos, PPI); Sas-hegy, Budapest (1 ♀, 16.07.2010., E. Botos, PPI); Sas-hegy, Budapest (1 ♀, 09.10.2010., E. Botos, PPI); Sas-hegy, Budapest (1 ♂, 07.10.1995., K. Bleicher, PPI); Gödöllő (1 ♂, 30.08.2012., G. Ambrus, HNHM Araneae-7703); Belsőbáránd (1 ♂, ?10.2010., Cs. Szinetár, HNHM Araneae-7704); Bugac (2 ♂, 24.09.2007., R. Gallé, HNHM Araneae-7705); Várpalota-Inota (1 ♀, 06.07.2014., G. Kovács, G. Vári, HNHM Araneae-7706), Ásotthalom (1 ♂, 20.10.2013., D. V. Nagy HNHM Araneae-7725), Mátyás-hegy, Budapest (2 ♂, 1933, G. Kolosváry, HNHM Araneae-2943).

*Eresus moravicus*. **Hungary:** Füzér, Castle hill (1 ♀, (juvenile at the time of collection), 07.10.2006., Cs. Szinetár, G. Kovács, HNHM Araneae-7707); Misina-hegy, Pécs (1 ♂, 22.04.2002., E. Vadkerti, HNHM Araneae-7708); Máriagyűd, Köves-máj (1 ♂, 08.05.2001, E. Vadkerti, HNHM Araneae -7709); Hárskút, Borostyán-hegy (1 ♂, 23.05.2011., L. Lajos, HNHM Araneae-7710); Misina-hegy, Pécs (3 ♂, 01.07.2011., E. Vadkerti, HNHM Araneae-7711); Felsőörs (1 ♂, 19.05.2011., M. Landy-Gyebnár, HNHM Araneae-7712); Cserkút (1 ♂, 26.05.2013., P. Kovács, HNHM Araneae-7713); Tatárszentgyörgy (4 ♂, 19.05.2013., H. Gyurkovics, G. Vári, HNHM Araneae-7714); Tatárszentgyörgy (2 ♀, 1 ♂, 19.05.2013., H. Gyurkovics, G. Vári, HNHM Araneae-7715); Tatárszentgyörgy (2 juv., 19.05.2013., H. Gyurkovics, G. Vári, HNHM Araneae-7716); Kelebia-Bácsborista (1 ♀, 1 ♂ (juvenile at the time of collection), 1 juv., 02.10.2011., H. Gyurkovics, G. Vári, HNHM Araneae-7717); Kelebia-Bácsborista (1 ♂, 30.05.2010., H. Gyurkovics, G. Vári, HNHM Araneae-7718); Misina-hegy, Pécs (3 ♀, 15.06.2012., G. Kovács, G. Vári, HNHM Araneae-7719); Szentgál, Tiszafás (1 ♂, 21.05.2012., M., Szabó, HNHM Araneae-7720); Kelebia-Bácsborista (1 ♂, 1 ♀, 06.06.2010., G. Vári, HNHM Araneae-7721); Kelebia-Bácsborista (1 ♂, 24.05.2010., H. Gyurkovics HNHM Araneae-7723); Kelebia-Bácsborista (2 ♀, 06.06.2010., G. Kovács, HNHM Araneae-7724); Várpalota, Várvölgy (1 ♂, subadult at the time of collecting, 14.11.2014., G. Kovács, HNHM Araneae-7725). **Austria:** Dürnstein, (1 ♀, 07.06.2012., W. Pfliegler, WPPC).

*Eresus sandaliatus*. **Denmark:** Clasonsborg (1 juv., 12.05.2004, J. Lissner, JLPC); Tranemose moor, Northwest Jutland (1 ♀, 02.10.2006, J. Lissner, JLPC); Heather at Gindeskov Krat, Aulum (1 ♂, 06.08.2004, J. Lissner, JLPC); Heather at Stovbaek Krat near Aulum (1 juv., 08.06.2004, J. Lissner, JLPC); Norlund, north of Hallundbaek Stream (1 ♂, 28.10.2011, J. Lissner, JLPC); near the Danish-German border (1 ♀, 05.08.2006, J. Lissner, JLPC); Vind Hede (1 ♀, 30.09.2008, J. Lissner, JLPC).
